# Synthesis and characterisation of brannerite compositions (U_0.9_Ce_0.1_)_1−*x*_M_*x*_Ti_2_O_6_ (M = Gd^3+^, Ca^2+^) for the immobilisation of MOX residues

**DOI:** 10.1039/c7ra11742f

**Published:** 2018-01-09

**Authors:** D. J. Bailey, M. C. Stennett, B. Ravel, D. Grolimund, N. C. Hyatt

**Affiliations:** Immobilisation Science Laboratory, Department of Materials Science and Engineering, University of Sheffield UK d.j.bailey@sheffield.ac.uk; National Institute of Standards and Technology 100 Bureau Drive Gaithersburg MD 20899 USA; Swiss Light Source, Paul Scherrer Institute Villigen 5232 Switzerland

## Abstract

A suite of uranium brannerites for the disposal of MOX residues, formulated (U_0.9_Ce_0.1_)_1−*x*_M_*x*_Ti_2_O_6_ (M = Ca^2+^ and/or Gd^3+^), were prepared using a mixed oxide route under oxidising, inert and reducing atmospheres (air, argon and H_2_/N_2_). Gd^3+^ was added to act as a neutron absorber in the final Pu bearing wasteform and Ce added to function as a structural analogue for Pu. X-ray powder diffraction of the synthesised specimens found that phase distribution was strongly affected by the processing atmosphere and Gd content. In all cases prototypical brannerite was formed, accompanied by different secondary phases dependent on processing atmosphere. Microstructural analysis (SEM) of the sintered samples confirmed the results of the X-ray powder diffraction. Bulk XANES found that Ti remained in the Ti^4+^ oxidation state whereas Ce was uniformly reduced to the Ce^3+^ oxidation state regardless of processing conditions or stoichiometry. Micro-focus XANES was used to determine U oxidation in the brannerite phase and showed that U oxidised to higher U oxidation states to charge compensate. It was concluded that the charge balance mechanism was a combination of U oxidation and A-site vacancies.

## Abstract 2

This work presents the synthesis and characterisation of novel brannerite ceramics designed for the disposal of mixed oxide fuel residues.

## Graphical abstract

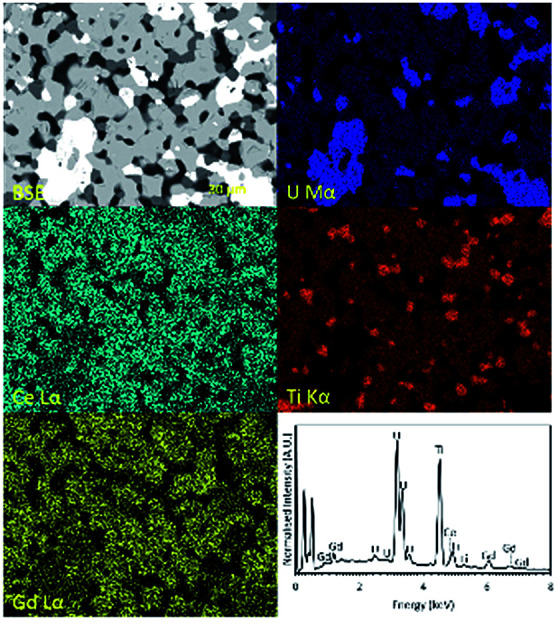

## 1 Introduction

Separated plutonium stockpiles, accumulated as a result of the continued reprocessing of spent nuclear fuel (SNF),^1^ present an ongoing challenge in terms of their safe, long-term management and also a potential proliferation risk.^2^ Consequently, reduction of accumulated stockpiles is highly desirable. Re-use of Pu in mixed oxide fuel (MOX), is an option currently favoured by the UK government.^1^ During production of mixed oxide fuels, residues rich in Pu and U are generated. Although the majority of MOX residues can be recycled and reused to make fuel, it is inevitable that some fraction will be uneconomic to recycle and will require immobilisation and disposal.

Brannerite (UTi_2_O_6_ ∼ 55 wt% U) is a titanate phase commonly found in uranium ore deposits^3^ and as an accessory phase in zirconolite and pyrochlore based ceramics designed for actinide disposition.^4,5^ Brannerite has a monoclinic crystal structure with space group *C*2/*m*, comprising layers of edge sharing TiO_6_ octahedra linked by octahedra with larger cations (U).^6^ Naturally occurring brannerites exhibit considerable chemical flexibility with elements such as Ca, Y, Pb, Ce and Th being incorporated on the U site and Fe, Si and Al substituting on the Ti site. However, they are commonly completely metamict due to accumulated α-recoil damage (critical dose 1–3 × 10^16^ α mg^−1^).^3,7^ Nevertheless, the presence of brannerite as a heavy mineral in alluvial sediments, after the host rock has been weathered, provides an indication of the long term durability of brannerite.^8^ Previous studies have shown that it is possible to synthesise Pu-bearing brannerites and also that the Pu analogue, Ce, adopts the brannerite structure.^4,9^ The production of a Pu-bearing wasteform may require the incorporation of neutron absorbing species, such as Gd or Hf, for criticality safety; these have been shown to form brannerite solid solutions with Pu by Vance *et al.*^4^

The synthesis of stoichiometric brannerite requires inert conditions.^10^ However, it is possible to stabilize the brannerite structure in air by the addition of dopants (Ca, La, Gd).^4,5,11–13^ This indicates that both processing conditions and target stoichiometry may affect brannerite formation.

In this study, the production of brannerites suitable for MOX disposal was investigated by synthesising brannerites with a range of conceptual waste loadings ((U_0.9_Ce_0.1_)_1−*x*_M_*x*_Ti_2_O_6_) under different atmospheres in an attempt to find a suitable baseline composition and processing conditions.

## 2 Materials and methods

### 2.1 Materials synthesis

Four different compositions of brannerite (Gd_0.1_U_0.81_Ce_0.09_Ti_2_O_6_, Gd_0.2_U_0.72_Ce_0.08_Ti_2_O_6_, Gd_0.25_U_0.675_Ce_0.075_Ti_2_O_6_ and Ca_0.1_Gd_0.1_U_0.72_Ce_0.08_Ti_2_O_6_) were synthesised by solid state reaction of component oxides and CaCO_3_ under oxidising, inert and reducing atmospheres (air, argon and 5% H_2_/N_2_). Gd was added to act as a neutron absorber in a final Pu-bearing wasteform and to stabilise higher U oxidation states (U^5+/6+^) in the brannerite structure by charge compensation (see below). Ce was used as a structural analogue for plutonium. MOX fuel compositions for light water reactors are envisaged to contain 5–10% Pu therefore the atomic ratio of U to Ce was maintained at 9 : 1 in all compositions as an approximation of the upper bounds of expected MOX fuel composition.^14^ Charge balancing of di- and trivalent cations (Ca^2+^ and Gd^3+^) was expected to occur *via* the oxidation of U^4+^ to higher oxidation states (U^5+^, U^6+^) as observed in previous studies.^4,5^

Reagents (UO_2_, CeO_2_, Gd_2_O_3_, TiO_2_ and CaCO_3_) were mixed with isopropanol to form a slurry and ball milled for five minutes at a frequency of 30 Hz using a Fritsch Pulverisette 23. The milled slurry was dried in an oven (95 °C) and a sample from each composition (∼0.6 g) reacted at 1300 °C for 12 hours under flowing air, argon or 5% H_2_/N_2_.

Sintered pellets were produced by uniaxially pressing reacted powders in a hardened steel die (6 mm diameter) and sintering under atmosphere (argon, air, 5% H_2_/N_2_) at 1320 °C for 12 hours.

### 2.2 Materials characterisation

Reacted powders were ground and characterised by powder X-ray diffraction with a Bruker D2 Phaser X-ray diffractometer using Cu Kα radiation. Cu Kβ radiation was filtered using a Ni foil. XRD data were processed using the Bruker DiffracEva software package.

Sintered pellets were characterised by scanning electron microscopy and energy dispersive X-ray spectroscopy (SEM-EDX) using a Hitachi TM3030 SEM equipped with a Bruker Quantax EDX. An accelerating voltage of 15 kV was used for imaging. EDX data were analysed using Bruker Quantax software. Sintered pellets were prepared for SEM analysis by mounting in cold setting resin and polishing with SiC paper and progressively finer diamond pastes to an optical finish (1 μm). Samples were sputter coated with carbon to reduce surface charging effects.

### 2.3 X-ray absorption near edge spectroscopy

Samples were characterised using X-ray absorption near edge spectroscopy (XANES) to determine the average oxidation state of U, Ce and Ti. Samples were investigated at the Ti K-edge (4966 eV), Ce L-III edge (5723 eV) and U L-III edge (17 166 eV) using a conventional XAS setup at the now decommissioned beamline X23A2, National Synchrotron Lightsource, Brookhaven National Laboratory. Samples were prepared for XAS analysis by homogenously mixing ground, reacted powders with polyethylene glycol and uniaxially pressing to form 13 mm diameter pellets of approximately one absorption length.

Samples were measured alongside standards of known oxidation states to allow the derivation of the average oxidation state of the element of interest. Ti and Ce edges were measured in fluorescence mode. Ti measurements were made alongside Ti^4+^, Ti^3+^, Ti^2+^ and Ti^(0)^ standards (TiO_2_, Ti_2_O_3_, TiO and Ti metal foil respectively) and Ce measurements were made alongside Ce^3+^ and Ce^4+^ standards (CePO_4_ and CeO_2_ respectively). The U L-III edge was measured in transmission alongside U^4+^, U^5+^ and U^6+^ standards: brannerite (U^4+^–UTi_2_O_6_), mixed brannerite (U^5+^–Y_0.5_U_0.5_Ti_2_O_6_) and calcium uranate (U^6+^–CaUO_4_). Y_0.5_U_0.5_Ti_2_O_6_ was produced according to the method described by James *et al.*^11^ The edge position was determined to be the maximum of the first derivative of the absorption spectrum, the average U oxidation state was determined by performing a linear regression of first derivative energies with respect to standards of known oxidation state.

Incident (*I*_0_) and transmitted (*I*_t_) X-ray intensities were measured using ion chambers, energy calibration was performed using XANES spectra measured with a reference ion chamber (*I*_r_) of a standard placed after the transmission ion chamber in the beam path. Fluorescence mode measurements were made using a four element vortex Si-drift detector. XANES spectra were measured from 30 eV below the edge of interest to 250 eV above. A Si (311) monochromator was used to tune the energy of incident photons giving an energy resolution of ±0.3 eV. Data reduction and XANES analysis were performed using the program Athena.^15,16^

### 2.4 Micro-focus X-ray absorption spectroscopy

To determine the U oxidation state exclusively in the brannerite phase, in the presence of accessory phases, samples were studied using micro-focus X-ray absorption spectroscopy at beamline X05LA, Swiss Light Source, Paul Scherrer Institute, Switzerland. Samples were mounted in cold setting resin before being thin-sectioned and mounted on a Spectrasil slide. For measurement, samples were mounted on a motorised *x*–*y*–*z* stage to allow scanning of the sample in the beam and a spot size of approximately 1 μm^2^ was used.

To aid in the selection of regions of interest, 30 μm^2^ fluorescence maps were produced by rastering the sample in the beam at an energy of 17 200 eV. X-ray fluorescence was measured using a Si drift detector (KETEK) mounted 45° to the incident beam. XRF maps were produced by windowing specific regions of the fluorescence spectrum corresponding to the emission lines of U, Gd and Ti. Points of interest for XANES study were then selected on the basis of relative intensities of Gd, Ti and U and knowledge of the respective phase assemblages and elemental partitioning of the samples from prior SEM-EDX analysis. Multiple XANES spectra per sample were measured from 30 eV below the U L-III edge to 250 eV above and then averaged, photon energy was tuned using a double crystal Si (111) monochromator. Again, data reduction and XANES analysis were performed using the program Athena.^15,16^ The average uranium oxidation state in the brannerite phase was determined by performing linear regression of the energy value at half the edge step with respect to standards of known oxidation state.

## 3 Results and discussion

### 3.1 Powder X-ray diffraction

**Fig. 1:**
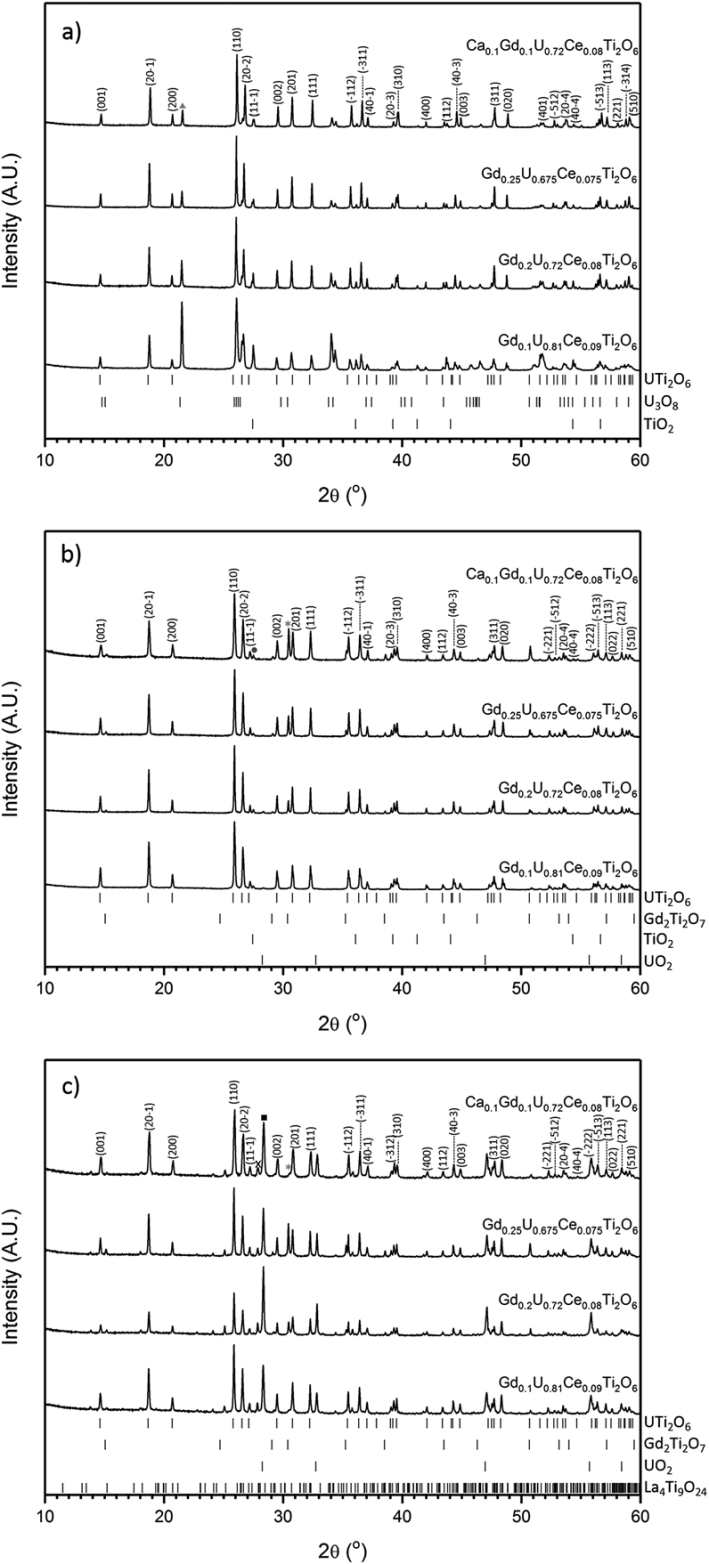
Powder X-ray diffraction results for compositions sintered under atmosphere: (a) air, (b) Ar and (c) 5% H_2_/N_2_. Major reflections of secondary phases are indicated by symbols: Triangle-U_3_O_8_; Circle-TiO_2_ (rutile); Square-UO_2_; Asterisk-Gd_2_Ti_2_O_7_ and X-La_4_Ti_9_O_24_. Reference patterns were obtained from the ICSD database (UTi_2_O_6_-ICSD 201342, Gd_2_Ti_2_O_7_-ICSD 167918, U_3_O_8_-ICSD 28137, TiO_2_ (rutile)-ICSD 33837, UO_2_-ICSD 246851, La_4_Ti_9_O_24_-ICSD 80052).

All samples produced were characterised by powder X-ray diffraction and sharp reflections indicative of prototypical brannerite were observed in all cases. Other, secondary phases, were observed dependent on processing atmosphere and composition. Samples sintered in air contained three phases: brannerite, U_3_O_8_ and rutile (TiO_2_); these results are in agreement with those reported by previous studies.^4,5,10,12,13^ As can be seen in Fig. 1a, as the level of Gd substitution increased the major peaks associated with U_3_O_8_ decreased, this indicates that the formation of the brannerite phase in air is stabilised by the addition of Gd.

Samples sintered in Ar were found to contain a mixture of prototypical brannerite, rutile and a pyrochlore phase, see Fig. 1b. Considering the solid solution limits quoted by James and Watson (2002) for brannerites in the series Gd_*x*_U_1−*x*_Ti_2_O_6_ produced under similar conditions, 0 < *x* < 0.45, these results are unexpected. Unlike samples sintered in air, increased substitution of Gd was seen to have a detrimental effect on the phase assemblage with the relative proportion of the pyrochlore phase showing a concomitant increase.

Samples sintered in 5% H_2_/N_2_, see Fig. 1c, were found to contain brannerite, UO_2_, a pyrochlore phase and a rare-earth rich titanate phase related to the La_4_Ti_9_O_24_ structure. A similar titanate phase has previously been found to form in CeTi_2_O_6_ ceramics when sintered in Ar and has also been observed in brannerite-based ceramics by Stefanovksy *et al.*^17^ As with samples produced in inert atmospheres, increased substitution of Gd led to an increase in the intensities attributed to the pyrochlore phase.

Although there was not significant variation in unit cell volume between different compositions produced in the same atmosphere; there is clear variation between samples produced in different atmospheres (Table 1). Samples produced in air were found to have the smallest unit cells and those produced in 5% H_2_/N_2_ the largest. Considering the ionic radii of U^4+^, U^5+^ and U^6+^ in octahedral coordination (0.89, 0.76 and 0.73 Å respectively), these results are consistent with the incorporation of more highly oxidised U in samples sintered in air and the retention of U^4+^ in samples produced in 5% H_2_/N_2_.

**Table 1:** Refined lattice parameters of synthesised brannerites

Nominal composition	Atmosphere	*a* (Å)	*b* (Å)	*c* (Å)	*β* (°)	*V* (Å^3^)
Gd_0.1_U_0.81_Ce_0.09_Ti_2_O_6_	Air	9.8172(2)	3.7390(1)	6.9196(2)	118.554(1)	253.997(2)
Gd_0.2_U_0.72_Ce_0.08_Ti_2_O_6_	Air	9.8210(2)	3.7370(1)	6.9101(1)	118.607(1)	253.611(2)
Gd_0.25_U_0.675_Ce_0.075_Ti_2_O_6_	Air	9.8207(1)	3.7371(1)	6.9099(1)	118.607(1)	253.599(1)
Ca_0.1_Gd_0.1_U_0.72_Ce_0.08_Ti_2_O_6_	Air	9.8145(2)	3.7346(1)	6.9027(1)	118.482(1)	253.007(2)
Gd_0.1_U_0.81_Ce_0.09_Ti_2_O_6_	Ar	9.8192(1)	3.7617(1)	6.9253(1)	118.807(1)	255.802(1)
Gd_0.2_U_0.72_Ce_0.08_Ti_2_O_6_	Ar	9.8215(2)	3.7612(1)	6.9240(1)	118.799(1)	255.779(2)
Gd_0.25_U_0.675_Ce_0.075_Ti_2_O_6_	Ar	9.8208(2)	3.7510(1)	6.9219(1)	118.797(1)	255.598(2)
Ca_0.1_Gd_0.1_U_0.72_Ce_0.08_Ti_2_O_6_	Ar	9.8210(6)	3.7660(2)	6.9293(4)	118.825(1)	256.284(6)
Gd_0.1_U_0.81_Ce_0.09_Ti_2_O_6_	5% H_2_/N_2_	9.8197(2)	3.7695(1)	6.9297(2)	118.864(1)	256.504(2)
Gd_0.2_U_0.72_Ce_0.08_Ti_2_O_6_	5% H_2_/N_2_	9.8286(5)	3.7702(2)	6.9324(4)	118.867(3)	256.884(5)
Gd_0.25_U_0.675_Ce_0.075_Ti_2_O_6_	5% H_2_/N_2_	9.8251(3)	3.7686(1)	6.9292(2)	118.867(1)	256.565(3)
Ca_0.1_Gd_0.1_U_0.72_Ce_0.08_Ti_2_O_6_	5% H_2_/N_2_	9.8192(2)	3.7693(1)	6.9294(1)	118.867(1)	256.468(2)

### 3.2 Scanning electron microscopy-energy dispersive X-ray spectroscopy (SEM-EDX)

**Fig. 2:**
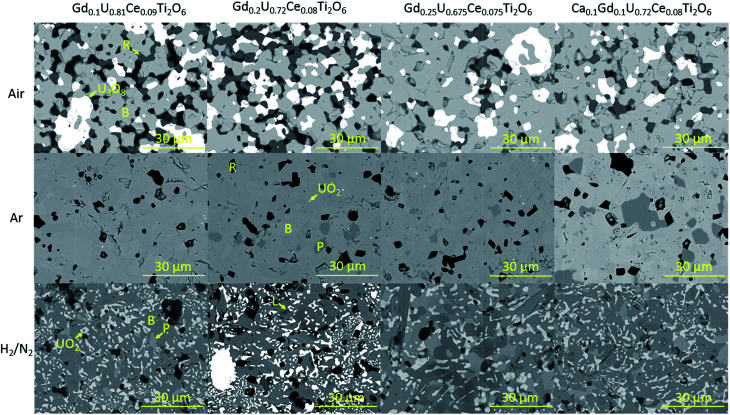
Representative backscattered electron images of sintered brannerites. B-brannerite, R-rutile, P-pyrochlore, L-Lan_4_Ti_9_O_24_.

Scanning electron microscopy was used to examine the phase distribution in sintered samples. In agreement with XRD data, brannerite was observed in all cases, with differing accessory phases dependent upon processing atmosphere. Representative backscattered electron images of sintered brannerites are shown in Fig. 2.

Three distinct phases were formed when samples were synthesised in air: brannerite, U_3_O_8_ and rutile (TiO_2_). Significant porosity was observed throughout all compositions, this is in agreement with the porosity observed by Vance *et al.* for samples synthesised under similar conditions.^4^

The phase distribution observed in samples sintered in argon was markedly different to that of samples sintered in air. In agreement with XRD results, there are several phases present within the samples; predominantly brannerite, rutile and pyrochlore with some retained UO_2_. As can be seen in Fig. 2, although there is no evidence of pyrochlore formation in the Gd_0.1_U_0.81_Ce_0.09_Ti_2_O_6_ composition, pyrochlore formation was observed for all other compositions. These results are in contrast to those reported by Vance *et al.* and James and Watson who found that substituted Gd formed a solid solution up to a limit of 0.45 formula units when Gd_*x*_U_1−*x*_Ti_2_O_6_ brannerites were produced in an inert atmosphere.^4,5^ Bailey *et al.* found that substitution of Gd in the system Gd_*x*_U_1−*x*_Ti_2_O_6_ led to the formation of a parasitic pyrochlore phase at a substitution level as low as 0.2 formula units,^12^ these results are consistent with those presented in this study.

Heat treatment of samples in a 5% H_2_/N_2_ atmosphere was found to result in the formation of brannerite, pyrochlore and a rare earth titanate phase along with the retention of UO_2_ throughout the samples.

**Fig. 3:**
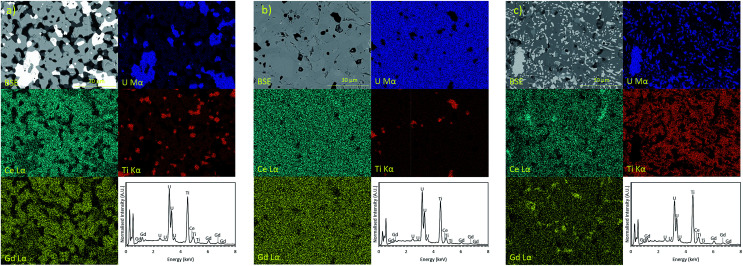
EDX spectra and elemental partitioning observed in Gd_0.1_U_0.81_Ce_0.09_Ti_2_O_6_ synthesised in: (a) air; (b) Argon and (c) 5% H_2_/N_2_.

From EDX analysis, it is evident that Gd and Ce are preferentially incorporated in the brannerite phase when samples are produced in an oxidising environment, Fig. 3a. Increased substitution of Gd may lead to the formation of single phase brannerite, however, the level of substitution required to achieve the desired phase assemblage would have a negative impact on the waste loading of a final wasteform.

Samples produced in an Ar atmosphere were found to exhibit different partitioning behaviour; as can be seen in Fig. 3b, Gd is clearly enriched in the pyrochlore phase and Ti is enriched in the rutile phase. Cerium does not clearly partition to either the brannerite or pyrochlore phase. However, the removal of Gd from the brannerite by the parasitic formation of pyrochlore may have a negative impact on the criticality performance of a final wasteform.

Samples synthesised in a reducing 5% H_2_/N_2_ atmosphere, Fig. 3c, exhibited similar elemental partitioning to those synthesised in an Ar atmosphere however; it is clear that a greater proportion of the U inventory is retained as UO_2_. Although there is some local enrichment visible in the EDX map, Ce does not show clear partitioning into any one phase.

### 3.3 Bulk X-ray absorption spectroscopy

**Fig. 4:**
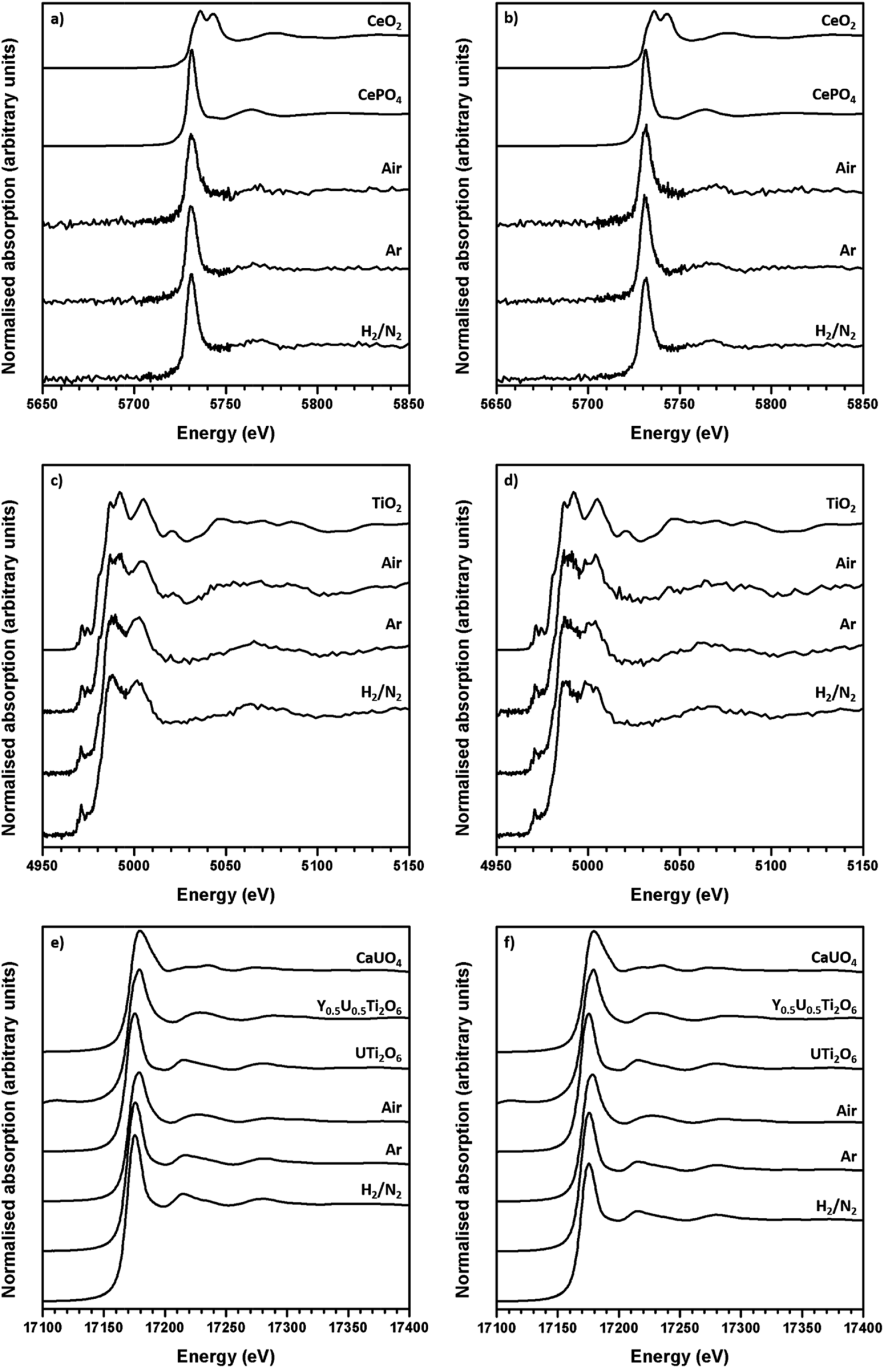
Representative XANES spectra of synthesised brannerites. (a) Ce L_III_ edge Gd_0.1_U_0.81_Ce_0.1_Ti_2_O_6_; (b) Ce L_III_ edge Ca_0.1_Gd_0.1_U_0.72_Ce_0.08_Ti_2_O_6_; (c) Ti K edge Gd_0.1_U_0.81_Ce_0.1_Ti_2_O_6_ and (d) Ti K edge Ca_0.1_Gd_0.1_U_0.72_Ce_0.08_Ti_2_O_6_; (e) U L_III_ edge Gd_0.1_U_0.81_Ce_0.1_Ti_2_O_6_ and (f) U L_III_ edge Ca_0.1_Gd_0.1_U_0.72_Ce_0.08_Ti_2_O_6_.

The average oxidation state of Ti, Ce and U in the synthesised brannerites were determined by X-ray absorption spectroscopy near edge spectroscopy (XANES). Fig. 4 shows representative Ce L-III, Ti K and U L-III edge XANES data.

**Fig. 5:**
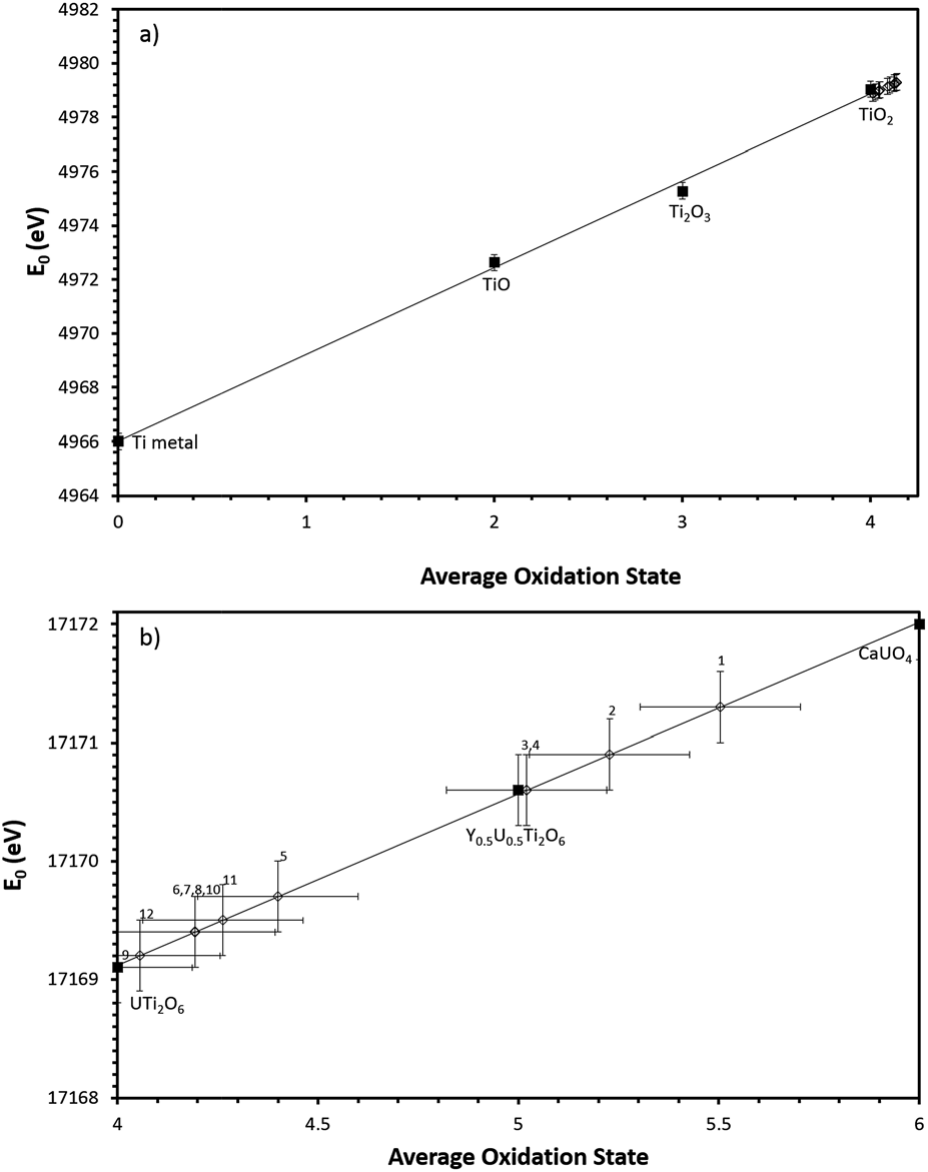
(a) Linear regression of Ti K edge *E*_0_ values with respect to Ti standards. (b) Linear regression of U L-III edge *E*_0_ values with respect to U standards. Numbers refer to corresponding composition no. in Table 2. Black squares denote standards, open diamonds denote samples.

Ti K-edge XANES data show that the predominant oxidation state of Ti in all samples is Ti^4+^, see Fig. 4c, d and 5a, as the white line positions (*E*_0_ = 4979.0 ± 0.3 eV) and pre-edge features are similar in character to the TiO_2_ standard.

This indicates that the oxidation state of Ti remains unchanged relative to the initial TiO_2_ precursor and is insensitive to both composition and processing atmosphere.

Ce L-III edge XANES data show that the position of the absorption edge and post-edge oscillations of Ce in synthesised brannerites are similar in character to the CePO_4_ standard (5725.0 eV). This indicates that the cerium has been reduced from Ce^4+^ to Ce^3+^ during synthesis. As can be seen, this reduction occurs regardless of processing atmosphere or composition and it is consistent with results of previous studies involving the substitution of Ce into the brannerite structure.^13,17^

As the Ti and Ce XANES data show, the oxidation states of Ce and Ti in the synthesised brannerites are insensitive to both processing atmosphere and stoichiometry. In contrast, it was found that the average U oxidation state was strongly affected by the processing atmosphere.

Samples synthesised in air exhibited an absorption edge and post-edge oscillations indicative of a mixed U^5+/6+^ oxidation state whereas samples synthesised under Ar or 5% H_2_/N_2_ possessed features indicative of mixed U^4+/5+^ oxidation states. Table 2 shows the average U oxidation state for the synthesised brannerites, as determined by linear regression with respect to standards (Fig. 5b). It is clear that processing atmosphere has a strong influence on the average oxidation state of U. However, comparison between different compositions processed in the same atmosphere does not reveal a strong compositional influence.

**Table 2:** Average U oxidation state of synthesised brannerites as determined by linear regression with respect to standards

Sample no.	Nominal composition	Atmosphere	Oxidation state
1	Gd_0.1_U_0.81_Ce_0.09_Ti_2_O_6_	Air	5.5 (±0.2)
2	Gd_0.2_U_0.72_Ce_0.08_Ti_2_O_6_	Air	5.2 (±0.2)
3	Gd_0.25_U_0.675_Ce_0.075_Ti_2_O_6_	Air	5.0 (±0.2)
4	Ca_0.1_Gd_0.1_U_0.72_Ce_0.08_Ti_2_O_6_	Air	5.0 (±0.2)
5	Gd_0.1_U_0.81_Ce_0.09_Ti_2_O_6_	Ar	4.4 (±0.2)
6	Gd_0.2_U_0.72_Ce_0.08_Ti_2_O_6_	Ar	4.2 (±0.2)
7	Gd_0.25_U_0.675_Ce_0.075_Ti_2_O_6_	Ar	4.2 (±0.2)
8	Ca_0.1_Gd_0.1_U_0.72_Ce_0.08_Ti_2_O_6_	Ar	4.2 (±0.2)
9	Gd_0.1_U_0.81_Ce_0.09_Ti_2_O_6_	5% H_2_/N_2_	4.0 (±0.2)
10	Gd_0.2_U_0.72_Ce_0.08_Ti_2_O_6_	5% H_2_/N_2_	4.2 (±0.2)
11	Gd_0.25_U_0.675_Ce_0.075_Ti_2_O_6_	5% H_2_/N_2_	4.3 (±0.2)
12	Ca_0.1_Gd_0.1_U_0.72_Ce_0.08_Ti_2_O_6_	5% H_2_/N_2_	4.1 (±0.2)

The presence of minor U-bearing accessory phases such as U_3_O_8_ and UO_2_ means that, although indicative, the bulk U oxidation state is not representative of the oxidation state of U in the brannerite phase. As a result, the oxidation state of U in the brannerite phase was subject to further investigation by micro-focus XAS.

### 3.4 Micro-focus X-ray absorption spectroscopy

**Fig. 6:**
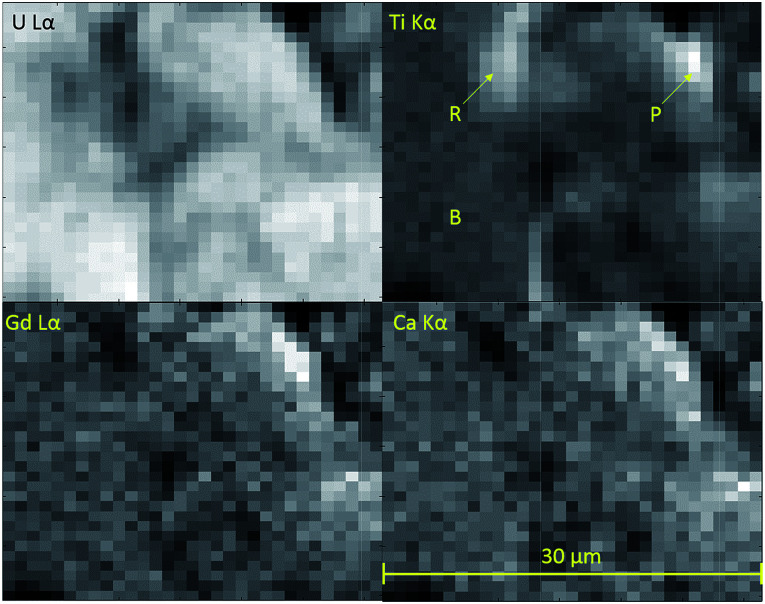
Fluorescence maps of Ca_0.1_Gd_0.1_U_0.72_Ce_0.08_Ti_2_O_6_ sintered in an argon atmosphere. Each pixel represents 1 μm^2^. Lighter colours indicate higher X-ray fluorescence intensity, darker colours indicate lower intensity. B-brannerite, P-pyrochlore and R-rutile.

Micro-focus X-ray spectroscopy was used to determine the oxidation state of U in the brannerite phase. XRF maps were used to determine which regions of the sample were brannerite and which were secondary phases, example maps are shown in Fig. 6. Rutile and pyrochlore phases were easily distinguished by a strong Ti Kα emission, together with Gd Lα or Ca Lα emission respectively. U_3_O_8_ and UO_2_ were distinguished by the absence of Gd Lα, Ca Lα and Ti Kα emission.

**Fig. 7:**
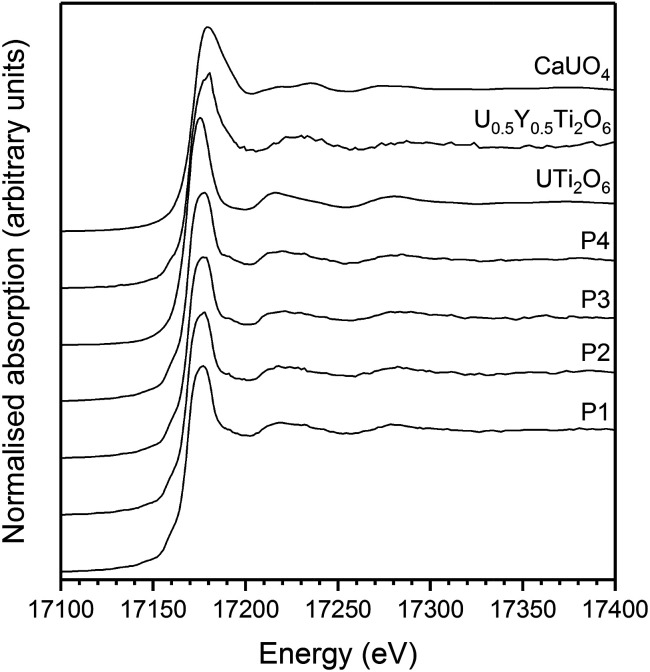
Example U L-III edge μ-focus XANES for Gd_0.1_U_0.81_Ce_0.09_Ti_2_O_6_ synthesised in argon. P1–4 indicate individual spectra taken from separate points.

U oxidation states, as determined by linear regression with respect to standards, are given in Table 3. Example micro-focus spectra are shown in Fig. 7. A general trend may be observed when comparing the effect of processing atmosphere on the U oxidation state; samples sintered in air had the highest U oxidation state, samples sintered in a reducing atmosphere had the lowest; and samples produced in inert conditions were intermediate between the two extremes. The oxidation of U to higher oxidation states was expected in order to compensate for the incorporation of Ca, Ce and Gd and is in agreement with the results reported by Vance *et al.*^4^ The relatively high oxidation state observed in samples sintered in air may be explained by the large amount of U_3_O_8_ present in the bulk of the sample: as Gd is incorporated in the brannerite phase and a substantial amount of U is incorporated in U_3_O_8_, the relative proportion of Gd to U in the brannerite phase is higher than in the target composition; consequently, U contained within the brannerite phase must oxidise further to compensate for the relative increase in Gd concentration.

**Table 3:** Oxidation state of uranium in the brannerite phase as found by micro-focus XAS and linear regression with respect to standards

Sample no.	Nominal composition	Atmosphere	Oxidation state
1	Gd_0.1_U_0.81_Ce_0.09_Ti_2_O_6_	Air	5.0 (±0.2)
2	Gd_0.2_U_0.72_Ce_0.08_Ti_2_O_6_	Air	4.6 (±0.2)
3	Gd_0.25_U_0.675_Ce_0.075_Ti_2_O_6_	Air	4.6 (±0.2)
4	Ca_0.1_Gd_0.1_U_0.72_Ce_0.08_Ti_2_O_6_	Air	5.2 (±0.2)
5	Gd_0.1_U_0.81_Ce_0.09_Ti_2_O_6_	Ar	4.5 (±0.2)
6	Gd_0.2_U_0.72_Ce_0.08_Ti_2_O_6_	Ar	4.5 (±0.2)
7	Gd_0.25_U_0.675_Ce_0.075_Ti_2_O_6_	Ar	4.5 (±0.2)
8	Ca_0.1_Gd_0.1_U_0.72_Ce_0.08_Ti_2_O_6_	Ar	4.6 (±0.2)
9	Gd_0.1_U_0.81_Ce_0.09_Ti_2_O_6_	5% H_2_/N_2_	4.4 (±0.2)
10	Gd_0.2_U_0.72_Ce_0.08_Ti_2_O_6_	5% H_2_/N_2_	4.1 (±0.2)
11	Gd_0.25_U_0.675_Ce_0.075_Ti_2_O_6_	5% H_2_/N_2_	4.5 (±0.2)
12	Ca_0.1_Gd_0.1_U_0.72_Ce_0.08_Ti_2_O_6_	5% H_2_/N_2_	4.4 (±0.2)

### 3.5 Charge balance mechanism

Combining the results of microfocus XANES with those of SEM-EDX and bulk XANES, a credible charge balance mechanism was derived. Assuming that oxygen is fully stoichiometric, the A-site is cation deficient and incorporates a small proportion of Ti. The charge balance mechanism is therefore hypothesised to be a combination of A-site vacancies and the oxidation of U^4+^ to higher oxidation states. Assuming this charge balance mechanism, the average composition closely follows that of the nominal, batched composition (Table 4) and is consistent with the secondary phases observed by SEM-EDX.

**Table 4:** Comparison of nominal composition and determined average composition of brannerites synthesised in argon atmosphere

Nominal composition	Determined average composition
Gd_0.1_U_0.81_Ce_0.09_Ti_2_O_6_	(Gd_0.09±0.01_U_0.76±0.04_Ce_0.04±0.01_Ti_0.04±0.03_□_0.07_)Ti_2.00±0.03_O_6_
Gd_0.2_U_0.72_Ce_0.08_Ti_2_O_6_	(Gd_0.17±0.01_U_0.71±0.03_Ce_0.05±0.01_Ti_0.04±0.03_□_0.03_)Ti_2.00±0.03_O_6_
Gd_0.25_U_0.675_Ce_0.075_Ti_2_O_6_	(Gd_0.21±0.03_U_0.66±0.03_Ce_0.04±0.01_Ti_0.06±0.06_□_0.03_)Ti_2.00±0.06_O_6_
Ca_0.1_Gd_0.1_U_0.72_Ce_0.08_Ti_2_O_6_	(Ca_0.02±0.02_Gd_0.09±0.02_U_0.79±0.02_Ce_0.03±0.01_□_0.07_)Ti_2.00±0.05_O_6_

## 4 Conclusions

Brannerite compositions have been synthesised under oxidising, inert and reducing atmospheres. Characterisation by XRD and SEM-EDX has shown that the final phase assemblage and elemental partitioning of brannerite wasteforms is affected by both processing atmosphere and stoichiometry. The most favourable phase assemblages were produced processing in an argon atmosphere. Increasing substitution of Gd and Ca was found to have a negative impact on the resultant phase assemblage of samples produced in inert atmospheres. The most favourable phase assemblage was produced for the target stoichiometry Gd_0.1_U_0.81_Ce_0.09_Ti_2_O_6_ when sintered in argon. Under these conditions Ce was incorporated into the brannerite phase indicating a promising route for immobilisation of expected MOX residue compositions. As the MOX compositions investigated in this study were at the upper bounds of expected Pu concentration for light water reactor fuels, it would be reasonable to assume that immobilisation of lower concentrations of Pu would not result in significant deviation from the outcomes of this study. Combined bulk and micro-focus XANES have established the oxidation states of Ti, Ce and U in synthesised brannerites. Combination of XANES and SEM-EDX data indicates a charge balance mechanism that uses a combination of U oxidation and A-site vacancies to achieve charge neutrality. It is clear that, in order to produce an optimised wasteform, careful consideration must be given to both the processing conditions and system stoichiometry.

## Conflicts of interest

There are no conflicts of interest to declare.

## Supplementary Material
