# Correction: Synthesis and characterisation of brannerite compositions (U_0.9_Ce_0.1_)_1−*x*_M_*x*_Ti_2_O_6_ (M = Gd^3+^, Ca^2+^) for the immobilisation of MOX residues

**DOI:** 10.1039/d0ra90131h

**Published:** 2021-01-19

**Authors:** D. J. Bailey, M. C. Stennett, B. Ravel, D. Grolimund, N. C. Hyatt

**Affiliations:** Immobilisation Science Laboratory, Department of Materials Science and Engineering, University of Sheffield UK d.j.bailey@sheffield.ac.uk; National Institute of Standards and Technology 100 Bureau Drive Gaithersburg MD 20899 USA; Swiss Light Source, Paul Scherrer Institute Villigen 5232 Switzerland

## Abstract

Correction for ‘Synthesis and characterisation of brannerite compositions (U_0.9_Ce_0.1_)_1−*x*_M_*x*_Ti_2_O_6_ (M = Gd^3+^, Ca^2+^) for the immobilisation of MOX residues’ by D. J. Bailey *et al*., *RSC Adv.*, 2018, **8**, 2092–2099. DOI: 10.1039/C7RA11742F.

The authors wish to correct an error in the unit cell volumes quoted in Table 1 of the original article. The corrected table is given below:

**Table 1:** Refined lattice parameters of synthesised brannerites

Composition	Atmosphere	*a* (Å)	*b* (Å)	*c* (Å)	*β* (°)	*V* (Å^3^)
Gd_0.1_U_0.81_Ce_0.09_Ti_2_O_6_	Air	9.8172(2)	3.7390(1)	6.9196(2)	118.554(1)	223.10(1)
Gd_0.2_U_0.72_Ce_0.08_Ti_2_O_6_	Air	9.8210(2)	3.7370(1)	6.9101(1)	118.607(1)	222.65(1)
Gd_0.25_U_0.675_Ce_0.075_Ti_2_O_6_	Air	9.8207(1)	3.7371(1)	6.9099(1)	118.607(1)	222.64(1)
Ca_0.1_Gd_0.1_U_0.72_Ce_0.08_Ti_2_O_6_	Air	9.8145(2)	3.7346(1)	6.9027(1)	118.482(1)	222.39(1)
Gd_0.1_U_0.81_Ce_0.09_Ti_2_O_6_	Ar	9.8192(1)	3.7617(1)	6.9253(1)	118.807(1)	224.14(1)
Gd_0.2_U_0.72_Ce_0.08_Ti_2_O_6_	Ar	9.8215(2)	3.7612(1)	6.9240(1)	118.799(1)	224.14(1)
Gd_0.25_U_0.675_Ce_0.075_Ti_2_O_6_	Ar	9.8208(2)	3.7600(1)	6.9219(1)	118.797(1)	223.99(1)
Ca_0.1_Gd_0.1_U_0.72_Ce_0.08_Ti_2_O_6_	Ar	9.8210(6)	3.7660(2)	6.9293(4)	118.825(1)	224.53(4)
Gd_0.1_U_0.81_Ce_0.09_Ti_2_O_6_	5% H_2_/N_2_	9.8197(2)	3.7695(1)	6.9297(2)	118.864(1)	224.64(1)
Gd_0.2_U_0.72_Ce_0.08_Ti_2_O_6_	5% H_2_/N_2_	9.8286(5)	3.7702(2)	6.9324(4)	118.867(3)	224.97(3)
Gd_0.25_U_0.675_Ce_0.075_Ti_2_O_6_	5% H_2_/N_2_	9.8251(3)	3.7686(1)	6.9292(2)	118.867(1)	224.69(2)
Ca_0.1_Gd_0.1_U_0.72_Ce_0.08_Ti_2_O_6_	5% H_2_/N_2_	9.8192(2)	3.7693(1)	6.9294(1)	118.867(1)	224.60(1)

Unit cell parameters were determined by a Le Bail refinement of XRD data using the Bruker Topas software package.

The subsequent discussion of the unit cell volume and the conclusions drawn in the manuscript are unaffected by this correction.

The Royal Society of Chemistry apologises for these errors and any consequent inconvenience to authors and readers.

## Supplementary Material

